# Underrepresentation of Elderly People in Randomised Controlled Trials. The Example of Trials of 4 Widely Prescribed Drugs

**DOI:** 10.1371/journal.pone.0033559

**Published:** 2012-03-30

**Authors:** Cécile Konrat, Isabelle Boutron, Ludovic Trinquart, Guy-Robert Auleley, Philippe Ricordeau, Philippe Ravaud

**Affiliations:** 1 INSERM U738, Paris, France; 2 Assistance Publique des Hôpitaux de Paris (AP-HP), Hôpital Hôtel Dieu, Centre d'Epidémiologie Clinique, Paris, France; 3 Université Paris Descartes, Faculté de Médecine, Paris, France; 4 French Cochrane Center, Paris, France; 5 Département de la Recherche Clinique, Institut National du Cancer (INCa), Paris, France; 6 Caisse nationale d'assurance maladie des travailleurs salariés (CNAMTS), Paris, France; Johns Hopkins Bloomberg School of Public Health, United States of America

## Abstract

**Background:**

We aimed to determine the representation of elderly people in published reports of randomized controlled trials (RCTs). We focused on trials of 4 medications—pioglitazone, rosuvastatin, risedronate, and valsartan—frequently used by elderly patients with chronic medical conditions.

**Methods and Findings:**

We selected all reports of RCTs indexed in PubMed from 1966 to April 2008 evaluating one of the 4 medications of interest. Estimates of the community-based “on-treatment” population were from a national health insurance database (SNIIR-AM) covering approximately 86% of the population in France. From this database, we evaluated data claims from January 2006 to December 2007 for 1,958,716 patients who received one of the medications of interest for more than 6 months. Of the 155 RCT reports selected, only 3 studies were exclusively of elderly patients (2 assessing valsartan; 1 risedronate). In only 4 of 37 reports (10.8%) for pioglitazone, 4 of 22 (18.2%) for risedronate, 3 of 29 (10.3%) for rosuvastatine and 9 of 67 (13.4%) for valsartan, the proportion of patients aged 65 or older was within or above that treated in clinical practice. In 62.2% of the reports for pioglitazone, 40.9% for risedronate, 37.9% for rosuvastatine, and 70.2% for valsartan, the proportion of patients aged 65 or older was lower than half that in the treated population. The representation of elderly people did not differ by publication date or sample size.

**Conclusions:**

Elderly patients are poorly represented in RCTs of drugs they are likely to receive.

## Introduction

Recent legislation incorporated comparative effectiveness research (CER) as a scientific mechanism to help improve health care [1]. CER aims to identify which treatments work in a real-world setting. This objective implies that the population included in clinical trials adequately represents the population treated in clinical practice.

Elderly people represent the fastest-growing segment of the population in western countries. In more developed regions of the world, people aged 65 or older represented 14% of the total population in 2000, and this proportion is anticipated to grow to 26% in 2050 [2]. The number of people aged 65 or older in the European Union will almost double over the next 50 year [3], [4]. Elderly people have a greater burden of chronic diseases and consume more medications than any other segment of the population. With increasing longevity, patients with chronic diseases and, consequently, the number of prescribed medications will increase [5], [6], [7].

The use of drugs in this population requires special consideration. Because of age-related changes in pharmocodynamics and pharmacokinetics, treatment effect sizes might differ. Moreover, because of co-morbidities and multiple medication regimens, elderly people are more likely to experience adverse drug reactions [8], [9]. Therefore, the US Food and Drug Administration (FDA) and the International Conference of Harmonisation (ICH E7) have recommended that elderly people be adequately represented in clinical trials for drugs likely to be used to treat diseases prevalent in this population. As well, pharmaceutical companies are required to include a geriatric-use subsection in documentation for their drugs.

Despite these recommendations, Van Spall et al. [10] highlighted that reports of randomized controlled trials (RCTs) published in major medical journals exhibited extensive unjustified exclusion criteria that may have affected the representation of elderly people in the RCTs. Further, studies in cardiology [11], [12], [13], [14], [15] and oncology [16], [17], [18] showed that elderly people were often undersampled in RCTs. However, in these studies, the proportion of elderly patients in trials evaluating a medication was not compared directly to the “on-treatment” population in clinical practice [14], [15], [16].

We aimed to compare the proportion of elderly people among the subjects enrolled in RCTs of drugs to the proportion of elderly people among corresponding patients treated in clinical practice.

## Methods

### Drugs Investigated in the Review

We were interested in medications commonly prescribed in primary care, orally administered, and only used for a limited number of different chronic conditions and in a homogenous population. We excluded medications having different indications according to patients' age.

A previous study [19] identified the most common chronic conditions of Medicare beneficiaries in primary care. They included cardiovascular disease (hypertension, chronic heart failure, stable angina, atrial fibrillation, hypercholesterolemia), metabolic diseases (diabetes mellitus), rheumatic diseases (osteoarthritis, osteoporosis) and lung diseases (chronic obstructive pulmonary disease). We did not consider the latter condition because medications used for this condition are usually administered by aerosol. We selected 4 medications specific to the corresponding chronic conditions: valsartan, an angiotensin receptor blocker indicated for hypertension, diabetic nephropathy and heart failure; rosuvastatin, a statin drug indicated for hypercholesterolemia and high cardiovascular risk; pioglitazone, a thiazolidinedione with hypoglycaemic action for type 2 diabetes mellitus; and risedronate, a third-generation biphosphonate for preventing and treating post-menopausal osteoporosis and treating glucocorticoid-induced osteoporosis.

### RCT Selection

We identified published reports of RCTs evaluating 1 of the 4 medications of interest by searching Medline via PubMed using the search terms “pioglitazone,” “rosuvastatin,” “risedronate,” and “valsartan,” with a limitation to RCTs published in French or English and no limitation on date of publication (RCTs published from 1966 up to and including April 2008).

The titles, abstracts, and full texts were assessed by one reviewer. Final selection was based on a review of full-text articles. We excluded reports of trials enrolling fewer than 30 patients in each treatment group, phase I and II trials, extended follow-up trials (i.e., follow-up of patients beyond the last outcome assessment), non-therapeutic trials (i.e., epidemiological studies), pathophysiological studies, ancillary reports of RCTs such as subgroup analyses, cost-effectiveness evaluations, systematic reviews, meta-analyses and trials assessing the organization of the healthcare system or interventions targeted to care providers.

We excluded subgroup analyses even if these analyses were performed for elderly patients. We made this choice because we wanted to avoid duplicate analyses of the same trial and we did not want to focus on subgroup analyses that may report biased results if not done appropriately. Overall, we excluded 3 articles reporting a subgroup analysis on elderly people; all were subgroup analyses of trials included in our sample.

### Characteristics of Reports

From the included reports, one author extracted data on type of journal (general medical journal or specialty journal), year of publication, funding sources (public, private, both, no funding, funding not reported or unclear), study design (parallel-group, cross-over, factorial design, other), number of groups, sample size, medication of interest (pioglitazone, rosuvastatin, risedronate, valsartan), type of control intervention (placebo, active treatment, usual care, other), single- or multicentre status, and trial location (number of countries and continents). As a quality control procedure, another author extracted a 10% random sample of the data. All discrepancies were discussed to obtain a consensus, with the 10% random sample considered the gold standard. Overall, the agreement between the author extracting the data for the whole sample and the gold standard was 98.3%.

### Representation of Elderly Subjects in Clinical Practice

To assess the age distribution of patients receiving medication in routine care, we obtained data from the major French national health insurance regime through the ”Système d'information inter-régime de l'Assurance maladie” (SNIIR-AM) database [20]. The SNIIR-AM covers approximately 86% of the population in France; approximately 53 million people. We selected data from January 1, 2006, to December 31, 2007, for patients who were at least 18 years old and taking one of the 4 medications of interest for more than 6 months, and then independently extracted data on patient age by medication of interest.

### Representation of Elderly Subjects in Selected Reports of RCTs

To evaluate the representation of elderly people in selected reports of clinical trials, one author collected data related to age (i.e., mean [SD] or median [25%–75% percentiles] and ranges). Lower and upper age limits as selection criteria were also collected.

Because the number of patients in the elderly category was rarely reported; for each RCT, we estimated the proportion of subjects aged 65 or older (and 75 or older) from the reported mean (SD) of age. We assumed that age followed a normal distribution. Because age was used as a selection criterion, the mean and SD were derived from truncated samples from the normal distribution (i.e., the sample was singly truncated when a lower age limit was reported or doubly truncated when both lower and upper age limits were reported) by the maximum likelihood method [21]. From these data, we derived the probability density function (p.d.f.) of the truncated distribution and estimated the proportion of subjects aged 65 or older (and 75 or older) by integrating this p.d.f. from 65 to infinity (and 75 to infinity). When age was not mentioned as a selection criterion, we assumed that a lower age limit of 18 was used. When reported, age ranges were used as truncation limits. Finally, 4 RCT reports contained discrepancies between the reported mean (SD) for age and the upper age limit reported as an eligibility criterion (the mean plus 1 SD lay above the upper limit for age as a selection criteria). In such cases, we did not consider the upper age limit.

### Statistical Analysis

Data are presented as numbers (%) for categorical variables and means (SD) or medians [25%–75% percentiles] for continuous variables.

To describe the representation of elderly in RCTs, we used funnel plots to plot (1) the mean age, (2) the estimated proportion of subjects aged 65 or older and (3) the estimated proportion of subjects aged 75 or older for each trial against the RCT sample size [22]. We calculated (1) the mean age, (2) the proportion of subjects aged 65 or older and (3) the proportion of subjects aged 75 or older in clinical practice from data in the SNIIR-AM database and plotted as horizontal lines with the associated 95% confidence interval, with a 0.05 probability of exceeding these limits for an RCT. For each medication, we counted how many RCTs had a proportion of subjects below and above the confidence interval limits and for how many RCTs the estimated proportion of patients aged 65 or older was less than half the SNIIR-AM value (i.e., relative difference between the estimated proportion of patients aged 65 or older and the SNIIR-AM proportion <−0.5). Finally, we compared these data for RCTs by publication year (2006–2007 vs. <2006) and sample size (≥500 vs <500).

Statistical analysis involved use of R software (R Foundation for Statistical Computing, Vienna, Austria, http://www.R-project.org) and STATA (StataCorp, College Station, Texas, USA).

## Results

### Characteristics of RCTs

**Figure 1:**
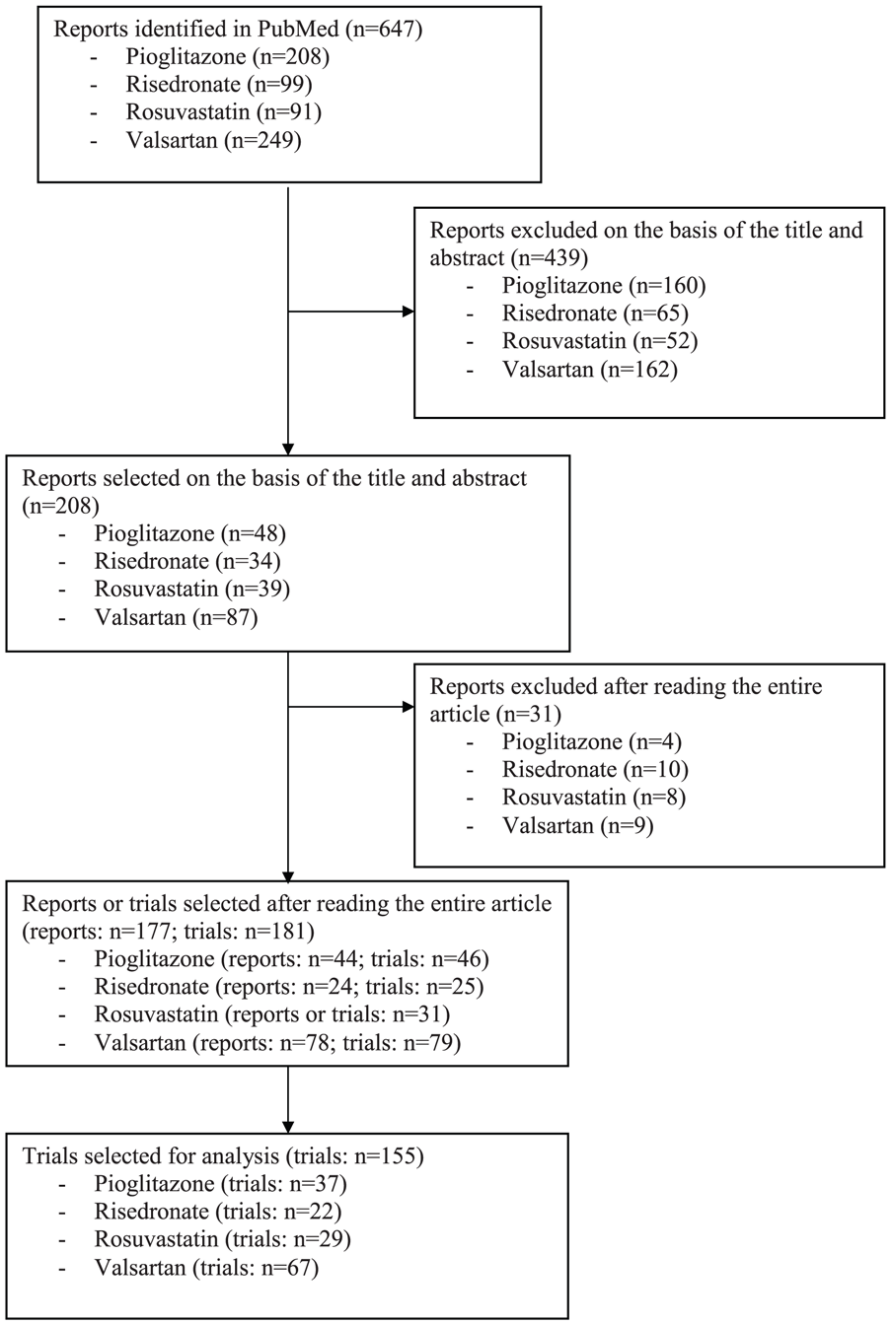
Flow chart of the number of published reports of randomized controlled trials identified and reviewed.

From the 647 reports of RCTs identified in PubMed investigating the 4 medications, 439 were excluded on the basis of the title or abstract and 31 after reading the full-text articles. Finally, we selected 177 articles. Among these, 4 reported results from 2 RCTs, which resulted in data for 181 RCTs. We selected for analysis the 155 (85.6%) trials reporting mean (SD) age or data on the age distribution, which allowed for calculation of mean (SD) age. Among the 155 trials selected for analysis: 37 assessed pioglitazone, 22 risedronate, 29 rosuvastatin and 67 valsartan (Figure 1). The characteristics of the 155 trials are summarized in Table 1. Private funding was the most commonly reported source of support, for 92 (59.3%) trials. The median sample size [25%–75% percentiles] was 328 [148–690].

**Table 1:** Characteristics of randomized controlled trials investigating drugs.

	AllN = 155	PioglitazoneN = 37	RisedronateN = 22	RosuvastatinN = 29	ValsartanN = 67
Funding source, n (%)					
Public agency	3 (1.9)	0	0	1 (3.4)	2 (3.0)
Private	92 (59.3)	25 (67.6)	11 (50.0)	23 (79.3)	33 (49.2)
Combination	7 (4.5)	0	4 (18.2)	0	3 (4.5)
Not reported	53 (34.2)	12 (32.4)	7 (31.8)	5 (17.2)	29 (43.3)
Sample size, median(25%–75% percentiles)	328(148–690)	281(186–566)	263(123–547)	509(293–871)	310(138–723)
Centres, n (%)					
Single-centre	21 (13.5)	5 (13.5)	4 (18.2)	2 (6.9)	10 (14.9)
Multicentre	123 (79.3)	31 (83.8)	16 (72.7)	27 (93.1)	49 (73.1)
Not reported	11 (7.1)	1 (2.7)	2 (9.1)	0	8 (11.9)
Continental Trial Location – n (%)†					
North America	56 (36.1)	14 (9.0)	6 (3.9)	19 (12.3)	17 (11.0)
Europe	61 (39.4)	16 (10.3)	7 (4.5)	13 (8.4)	25 (16.1)
Central and South America	9 (5.8)	1 (0.6)	1 (0.6)	2 (1.3)	5 (3.2)
Asia/Oceania	20 (12.9)	7 (4.5)	6 (3.9)	1 (0.6)	6 (3.9)
Africa	3 (1.9)	1 (0.6)	0	0	2 (1.3)
Not reported	38 (24.5)	9 (5.8)	4 (2.6)	2 (1.3)	23 (14.8)

† Categories not mutually exclusive.

The proportion of patients aged 65 or older was given for 20 (11.1%) trials.

Overall, 75% of the trials had at least 1 centre located in North America or Europe; and the location was not reported in 24% of the reports. Only 4 trials did not involve any centres from high-income countries.

### Representation of Elderly Patients in RCTs

From January 1, 2006 to December 31, 2007, 1,958,716 patients recorded in the SNIIR-AM database had a prescription for at least 1 of the 4 medications of interest for more than 6 months and were aged 18 or older: 124,987 for pioglitazone, 309,084 for risedronate, 744,665 for rosuvastatin, and 893,422 for valsartan.

The mean (SD) age of patients was 62.9 (11.6) for patients receiving pioglitazone, 71.2 (11.5) for risedronate, 62.3 (12.1) for rosuvastatin, and 66.1 (12.8) for valsartan. The number (%) of patients aged 65 or older was 55,487 (44.4%) for pioglitazone, 224,370 (72.6%) for risedronate, 319,204 (42.9%) for rosuvastatin, and 490,968 (55.0%) for valsartan. The number (%) of patients aged 75 or older was 21,773 (17.4%) for pioglitazone, 135,369 (43.8%) for risedronate, 129,618 (17.4%) for rosuvastatin, and 260,562 (29.2%) for valsartan. Only 3 trials exclusively included elderly patients (2 assessing valsartan, 1 assessing risedronate).

**Figure 2:**
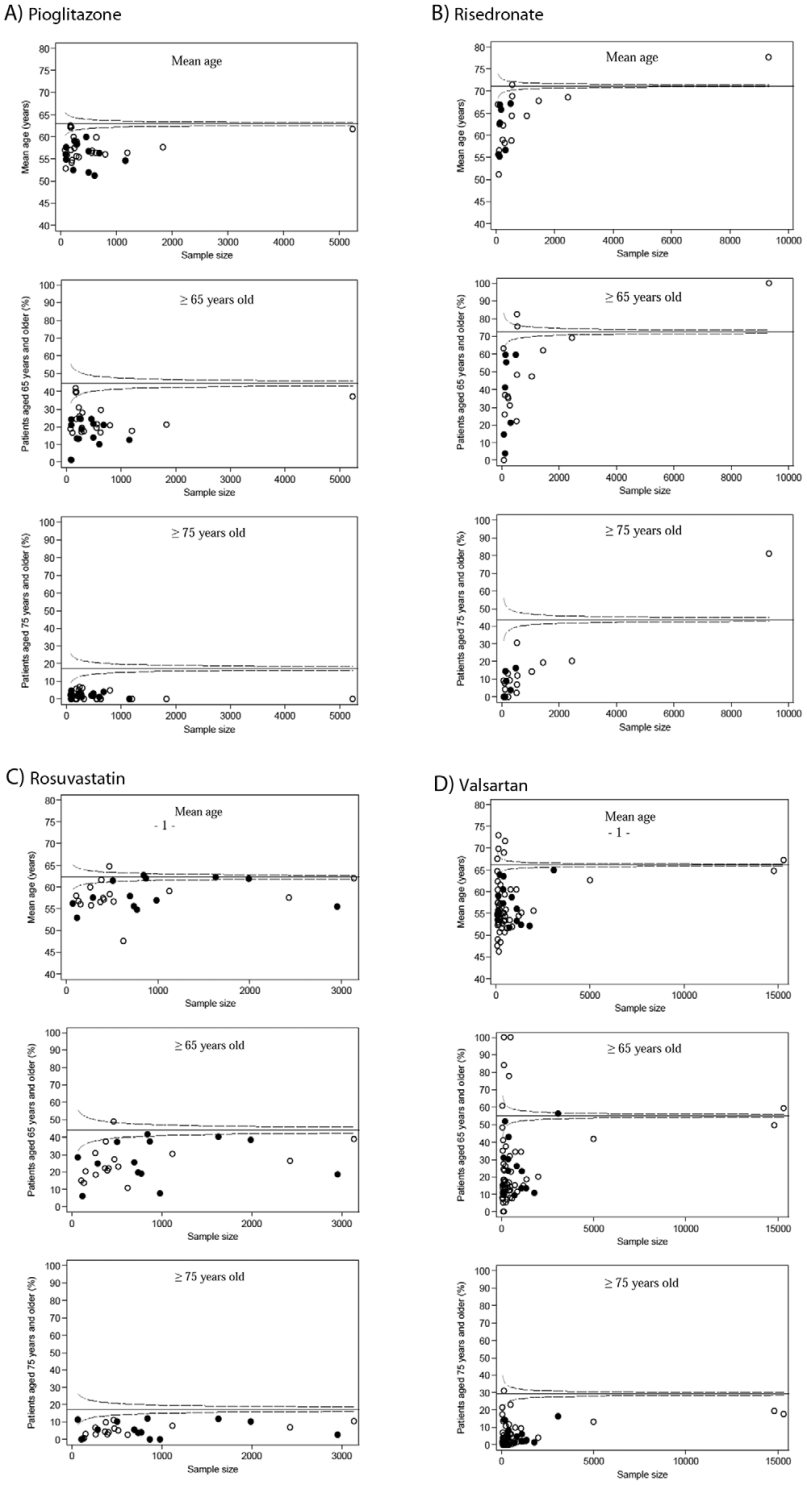
Funnel plots displaying (1) mean age, (2) estimated proportion of subjects aged 65 or older and (3) estimated proportion of subjects aged 75 or older plotted for each trial against the trial's sample size. (1) Mean age, (2) proportion of subjects aged 65 or older and (3) proportion of subjects aged 75 or older in clinical practice calculated from the SNIIR-AM database were plotted as horizontal lines (plain lines) with the corresponding 95% confidence interval (95% CI; dashed lines). For each medication, the plot shows how many RCTs had a proportion of older subjects below or above the 95% CI limits (0.05 probability of exceeding these limits) (i.e., RCTs with significantly lower or higher representation of elderly people as compared with the community-based “on-treatment” population). The plot allows for assessing how the proportion of elderly people varies with trial size and time. White dots represent older trials (i.e., trial reports published before 2006) and black dots represent recent trials (i.e. trial reports published in or after 2006).

As shown in Figure 2, elderly people were poorly represented in most trials. Only 4 of 37 reports for pioglitazone (10.8%), 4 of 22 (18.2%) for risedronate, 3 of 29 (10.3%) for rosuvastatine and 9 of 67 (13.4%) for valsartan investigated a proportion of patients aged 65 or older that was within or above the proportion treated in clinical practice. Similarly, no RCT had a proportion of patients aged 75 or older for pioglitazone and rosuvastatine, and only 1/25 (4%) for risedronate and 2/67 (3.0%) for valsartan. However, as shown in Figure 2, few trials of risedronate and valsartan with an adequate representation of elderly included large numbers of patients.

**Figure 3:**
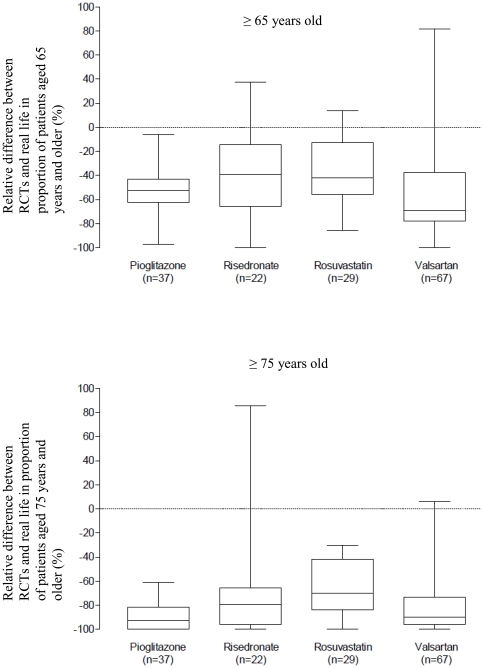
Box plots for the relative difference between the randomized controlled trials (RCTs) and clinical practice in proportion of patients aged 65 years and older and 75 years and older. Boxes represent median values (horizontal rule) with 25th and 75th percentiles (top and bottom of box). Error bars represent the 10th and 90th percentiles.

The relative differences between the trial and clinical practice proportions of patients aged 65 or older and 75 or older are in Figure 3. Overall, the proportion of patients 65 or older in the trial reports was lower than half the proportion in the treated population in 62.2% of reports for pioglitazone, 40.9% for risedronate, 37.9% for rosuvastatine, and 70.2% for valsartan (table 2).

**Table 2:** Published trials with a low representation of patients aged 65 and older, defined as trials including less than half of the proportion of elderly people in the treated population.

	All trials	Recent trials(≥2006)	Older trials(<2006)	p value	Low sample size (<500)	High sample size (≥500)	p value^1^
**Pioglitazone N = 37**	23 (62)	9/12 (75)	14/25 (56)	0.29	12/24 (50)	11/13 (85)	0.07
**Risedronate N = 22**	9 (41)	3/7 (43)	6/15 (40)	0.90	8/14 (57)	1/8 (12)	0.07
**Rosuvastatin N = 29**	11 (38)	5/13 (38)	6/16 (40)	0.96	6/14 (43)	5/15 (33)	0.71
**Valsartan N = 67**	47 (70)	10/15 (67)	37/52 (71)	0.74	34/47 (72)	13/20 (65)	0.57

Data are number (%).

1 Fisher exact test.

We performed a sensitivity analysis excluding the 4 trials that did not involve any high-income countries (i.e., 2 conducted in Asia/Oceania only and 2 conducted in Central and South America only). All these trials assessed valsartan. The sensitivity analysis yielded consistent results. In fact, the proportion of patients aged 65 or older was within or above that treated in clinical practice for 14.3% of trials (9/63) and was lower than half that in the treated population for 68.3% of trials (43/63).

The representation of elderly people did not systematically differ by publication date or sample size (Table 2).

## Discussion

This study assessed the representation of elderly patients in reports of RCTs evaluating 4 medications routinely used for elderly patients. Only 13% and 2% of the articles reported a proportion of patients aged 65 or older and 75 or older, respectively, that was within or above the proportion treated in clinical practice. Further, the proportion of patients aged 65 and older was lower than half the proportion in the treated population in 58% of the reports.

To our knowledge, this is the first study to directly compare the proportion of elderly patients included in trials of medications to that of a representative population receiving these medications in clinical practice. In fact, we used the SNIIR-AM database, a large healthcare insurance database covering all geographic areas in France and representing a broad segment of the population in terms of socioeconomic status, occupation and other demographics. The SNIIR-AM covers approximately 86% of the population in France. Although no study has compared the representativeness of SNIIR-AM patients to the general French population, the 2 populations are not expected to differ [23].

Our results are consistent with those of previous studies [24],[25]. By studying the reported age ranges or upper age limits as selection criteria, some studies showed the exclusion of elderly people from RCTs of arthritis [26], [27] or statins [11]. Similarly, Van Spall et al. [10] found a high rate of poorly justified exclusion criteria in trials and that most of the unjustified exclusions were related to age and comorbidities. A study of ongoing trials of heart failure showed that 43% of the trials had one or more poorly justified criteria that could limit the inclusion of older subjects [28].

Underenrolment of elderly subjects in trials can have significant safety implications when the results of a trial are applied to management for elderly subjects in routine clinical practice. In fact, older patients can have unexpected treatment response in terms of comorbidities and comedications [29], [30] For example, in a recent study [31], the addition of spironolactone to the standard treatment for heart failure was considered efficient and safe. However, once included in clinical practice, spironolactone added to standard therapy for heart failure unexpectedly resulted in a substantial increase in morbidity and mortality in elderly patients [32]. Moreover, the lack of strong evidence for the use of new therapies for elderly patients may contribute to poorly justified prescriptions, because many physicians extrapolate results from RCTs of adult patients to their practice with elderly patients, and to the high rate of underuse of evidence-based therapies (i.e., a drug that is not prescribed to treat or prevent a specific condition, despite no contraindication) that has been extensively recognized in this population [29], [33], [34], [35], [36].

The need to improve the representation of elderly in clinical trials has been recognized by important stakeholders. The CER initiative identified elderly people as a priority population. The list of the 100 highest priority research questions identified for CER contains several priority topics affecting elderly people or dedicated only to elderly people [37]. Recently, the European Union leaders brought together governmental officials, industry, health professionals, and other stakeholders from across Europe to improve the translation of research in practice [4]. Among the most common problems identified to translate research into practice was the lack of clear, accessible evidence about new technology for elderly people.

Improving the representation of elderly patients in trials is challenging because of the multiplicity of factors contributing to their exclusion. Protocol restrictions with exclusion criteria on age, co-medications and co-morbidities are an important limitation responsible for the exclusion of elderly people from trials. Further, the perceived difficulties by investigators related to screening and retaining elderly patients in trials could be a barrier to their inclusion in RCTs [38], [39], [40]. In fact, convenience and study efficiency may explain the exclusion of elderly people because a large number of older patients must be screened before one study participant is enrolled [15].

Our study contains some limitations. First, we selected RCTs indexed in PubMed up to April 2008 and we obtained data for the patients actually treated from January 2006 to December 2007. However, we have few reasons to believe that the results may have changed in 2012. Second, we examined only published reports of RCTs, which leaves open the possibility of publication bias in our results. However, published reports of RCTs are a source of medical information that physicians can easily consult, and they may have the greatest impact on prescription in clinical practice. As well, we focused on 4 currently prescribed medications, so the results of the study may not be generalized to other drugs. The SNIIR-AM database is a French database and the generalizability of our results to other countries may be debatable. Of note, pioglitazone was one of the drugs selected. Since our study, important information on risks of thiazolidinediones – increased risk of bladder cancer and cardiovascular problems – has been revealed and has changed prescribing practices. However, this situation should not affect our results because we chose articles published before this withdrawal [41]. Further, we focused on a definition of elderly people based on age. Information was not routinely available on the proportion of elderly in these trials. We estimated it from the average and dispersion values based on a normality distribution. We cannot exclude that in some trials, mean age departed from this assumption. However, we took it into account as much as possible using truncated normal distributions. Besides, an evaluation of the representation of elderly people in trials should distinguish between fit and frail elderly people. In our study, a single reviewer extracted the data. However, a quality assurance procedure consisting of a second reviewer extracting the data for 10% of the selected articles confirmed the appropriate quality of the data. Finally, we selected the trials whatever the location of the study. All but 4 trials involved centers from high-income countries. We performed a sensitivity analysis excluding these studies and the results were similar.

In light of the results of this study, the representativeness of elderly subjects in RCTs still needs improvement. Factors contributing to underrepresentation have been extensively discussed and can be overcome. Implementing more incentive approaches should be considered. A combination of laws and regulations could lead to substantial increases in the number of clinical trials of medications that elderly subjects frequently use, as was demonstrated for paediatric clinical trials [42], [43]. Querying databases similar to the SNIIR-AM could help set *a priori* goals for recruiting elderly people in RCTs.
